# Plasmonic Molecular Nanohybrids—Spectral Dependence of Fluorescence Quenching

**DOI:** 10.3390/ijms13011018

**Published:** 2012-01-18

**Authors:** Maria Olejnik, Łukasz Bujak, Sebastian Mackowski

**Affiliations:** Optics of Hybrid Nanostructures Group, Institute of Physics, Nicolaus Copernicus University, Grudziadzka 5/7, 87-100 Torun, Poland; E-Mails: marysia8@doktorant.umk.pl (M.O.); bujak@fizyka.umk.pl (L.B.)

**Keywords:** plasmon excitation, fluorescence, conjugation, gold nanoparticle, organic dye

## Abstract

We demonstrate strong spectral dependence of the efficiency of fluorescence quenching in molecular systems composed of organic dyes and gold nanoparticles. In order to probe the coupling with metallic nanoparticles we use dyes with varied spectral overlap between the plasmon resonance and their absorption. Hybrid molecular structures were obtained via conjugation of metallic nanoparticles with the dyes using biotin-streptavidin linkage. For dyes featuring absorption above the plasmon excitation in gold nanoparticles, laser excitation induces minute changes in the fluorescence intensity and its lifetime for both conjugated and non-conjugated mixtures, which are the reference. In contrast, when the absorption of the dye overlaps with the plasmon resonance, the effect is quite dramatic, reaching 85% and 95% fluorescence quenching for non-conjugated and conjugated mixtures, respectively. The degree of fluorescence quenching strongly depends upon the concentration of metallic nanoparticles. Importantly, the origin of the fluorescence quenching is different in the case of the conjugated mixture, as evidenced by time-resolved fluorescence. For conjugated mixtures of dyes resonant with plasmon, excitation features two-exponential decay. This is in contrast to the single exponential decay measured for the off-resonant configuration. The results provide valuable insight into spectral dependence of the fluorescence quenching in molecular assemblies involving organic dyes and metallic nanoparticles.

## 1. Introduction

Plasmon excitation, the result of collective electron oscillation in metallic nanoparticles, has been used for more than a decade to control the optical properties of fluorophores. The ability to control and very often dramatically modify absorption or fluorescence of a molecule by placing a metallic nanoparticle in its vicinity owes to strong localization of the electromagnetic field by the latter. There are three key parameters that determine the strength of the interaction [1]. First of all, the relation between the energy of plasmon excitation and the optical properties of a fluorophore implies whether the emission or the absorption of the fluorophore is enhanced. In order to observe strong fluorescence radiative rate enhancement the plasmon resonance should overlap with the emission spectrum of the fluorophore [2]. Analogous relation has to be conserved for predominant enhancement of the absorption [3]. Secondly, the distance between the fluorophore and the metallic nanoparticle determines whether the fluorescence is enhanced due to local electromagnetic field created by the metallic nanoparticle or, alternatively, if the energy is efficiently transferred from the fluorophore to the metallic nanoparticle, which would result in fluorescence quenching [4,5]. Last but not least, the relative orientation of the fluorophore and the metallic nanoparticle to the laser excitation could contribute to the net effect measured for an assembly comprising fluorophores and metallic nanoparticles. This parameter has proven to be the hardest to control. The benchmark experiment that evaluates all these parameters and their impact on the strength of plasmon-induced interaction between a single metallic nanoparticle and a single fluorophore has been reported by Anger *et al.* [2]. The metallic nanoparticle was placed at the end of the tip and precisely positioned above the emitter at distances between 0 and 60 nm. Upon changing the separation between the two particles the fluorescence intensity was measured. It has been found that there exists an optimal distance, typically in the range of 10 to 20 nm, for which the enhancement of the fluorescence intensity is the strongest. For smaller distances, the quenching was observed while for larger distances there was essentially no effect upon the fluorescence intensity. Similar qualitative behavior has been observed for a few other hybrid nanostructures comprising metallic nanoparticles and either semiconductor quantum dots [6–8], organic molecules [9–11], or natural photosynthetic complexes [12–15].

Several architectures that allow the study of interactions between plasmon excitations and fluorophores have been proposed and demonstrated in addition to the highly sophisticated strategy described in [2]. The most straightforward one is a mixture of both components in solution [16]. In this case there is no robust control of the separation between the nanoparticles and fluorophores or of their relative orientation. It has been frequently shown that mixing fluorophores with metallic nanoparticles directly in solution leads to efficient quenching of the fluorescence [5,16]. In order to more accurately define the distance between the fluorophores and metallic nanoparticles, bioconjugation approach has been applied [6], in which both metallic nanoparticles and fluorophores are functionalized with proteins or functional groups characterized with high affinity for binding. One of the most prominent examples is the biotin-streptavidin linker used for attaching gold or silver nanoparticles to semiconductor nanoparticles [6]. In the case of bioconjugation, some degree of separation control has been demonstrated by using polymer chains as intermediates [17]. Another strategy is to fabricate a layered structure, in which a thin layer of metallic nanoparticles is separated from the fluorophores by a dielectric layer with controlled thickness [3,14]. The experiments carried out for such structures, where the distance can be precisely controlled, have mirrored the dependence of the fluorescence intensity upon the distance as measured for a single molecule architecture [14].

In this work we focus on a molecular assembly obtained in solution using bioconjugation approach with biotin-streptavidin linker. The structure consists of gold spherical nanoparticles conjugated with Atto organic dyes. While the plasmon resonance of the nanoparticles appears approximately at 530 nm, the absorption maximum of the dyes varied between 488 nm and 550 nm. In this way we can study the spectral dependence of the plasmon-induced effects upon the fluorescence properties of the dyes. In the case of reference sample, where organic dyes were mixed directly with non-functionalized gold nanoparticles we observe a modest reduction in fluorescence intensity, with the strongest effect seen for Atto 550. These changes in intensity were not accompanied with modifications of fluorescence transients. In contrast, when gold nanoparticles are functionalized with streptavidin, thus enabling conjugation, the fluorescence quenching is much stronger, reaching up to 95%. The degree of the quenching depends critically upon the overlap between the plasmon resonance and absorption/emission of the dye molecules: the larger the overlap the stronger the effect of quenching. For streptavidin-functionalized gold nanoparticles the fluorescence decays of Atto dyes feature characteristic behavior. While the transients measured for Atto 488—Au conjugate show no dependence upon the concentration of gold nanoparticles, the quenching—and thus rapid shortening of the fluorescence lifetime—in the case of Atto 550 is almost instantaneous. The results indicate that a bioconjugate is formed in a solution and that plasmon-induced effects depend upon the relation between the optical properties of the fluorophore and metallic nanoparticles. These observations will be important for designing novel molecular sensors based upon plasmonic interactions [17].

## 2. Results and Discussion

### 2.1. Spectroscopic Characterization

**Figure 1:**
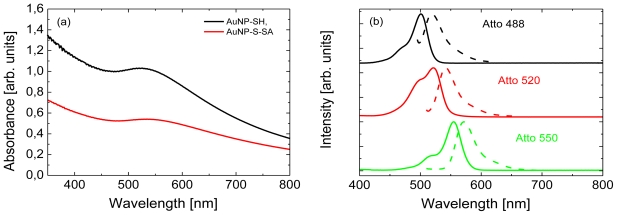
(**a**) Absorption spectrum of gold nanoparticles upon functionalization with streptavidin (red) compared to bare nanoparticles. The sepctra are not scaled. (**b**) Absorption and fluorescence spectra of the Atto dyes: Atto 488 (black), Atto 520 (red), and Atto 550 (green).

In Figure 1 we present the optical spectra of all the components used for assembling molecular hybrid nanostructures. The absorption spectrum of the gold nanoparticles in solution (Figure 1a) features prominent plasmon resonance at 521 nm. Upon attachment of streptavidin to the nanoparticles we observe measurable shift of the resonance towards lower energies (537 nm, red). It is well known that such a shift takes place due to change in local dielectric constant due to attachment of the protein [6]. Therefore, it may suggest that streptavidin is indeed attached to gold nanoparticles. In Figure 1b we show fluorescence and absorption of three Atto dyes used for conjugation with gold nanoparticles: Atto 488 (black), Atto 520 (red), and Atto 550 (green). Both the absorption and the fluorescence spectra are very similar across all three dyes except for the higher intensity of the high-energy shoulder in the absorption spectrum of Atto 520. The maxima of absorption appear at 500 nm, 520 nm and 550 nm for these molecules, with corresponding fluorescence maxima at 518 nm, 540 nmm and 570 nm. By comparing these data with the spectrum measured for gold nanoparticles functionalized with streptavidin, we see that in the case of Atto 488 the absorption as well as emission are located above the plasmon energy of the nanoparticles. The spectra of Atto 520 match much better with the plasmon resonance of the nanoparticles than for Atto 488, while maxima of emission and absorption of Atto 550 are almost perfectly associated with the plasmon resonance. The set of fluorescent dyes gives us not only an opportunity to test the conjugation procedure but also to study plasmon-induced changes in qualitatively different spectral overlap configurations.

### 2.2. Optical Properties of Atto-Au NP Conjugates

**Figure 2:**
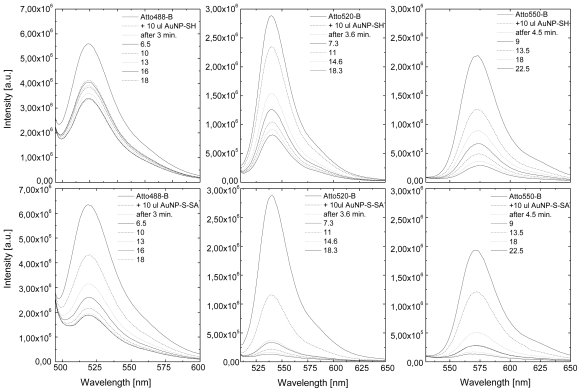
(**top row**) Fluorescence spectra measured for Atto dyes mixed directly with non-functionalized gold nanoparticles. (**bottom row**). Fluorescence spectra measured for Atto dyes mixed with gold nanoparticles functionalized with streptavidin. Sequences were taken over approximately 20 min. for each mixture. Excitation energy of 485 nm was used.

Fluorescence spectra of Atto dyes mixed with gold nanoparticles are shown in Figure 2. For all measurements the excitation wavelength was 485 nm. The experiments were carried out on mixtures containing Atto dye and non-functionalized gold nanoparticles (upper row) and gold nanoparticles with streptavidin attached (lower row). The emission was monitored through over 20 min. after mixing the components in a quvette. The measurements were repeated several times for each preparation and the results were very similar to the ones displayed in Figure 2. For all sample preparations we observe a decrease in fluorescence intensity as a function of time. We note that, for pure Atto dyes in aqueous solution, the effect of photobleaching is much less compared to the fluorescence quenching observed for Atto-Au nanoparticle mixtures and it is similar to all three dyes. The comparison for Atto dyes with different absorption/fluorescence maxima mixed with non-functionzalized gold nanoparticles indicates that the effect is the strongest for the Atto 550, the one that features the highest absorption overlap with the plasmon excitation in metallic nanoparticles. This quenching results from random movement of both the nanoparticles and fluorophores in the solution. The percentage of molecules quenched after approximately 20 min. from the start of the experiment was determined to be 41%, 73%, and 85% for Atto 488, Atto 520, and Atto 550, respectively. Repeating the same experiment with the same concentrations, but with the gold nanoparticles functionalized with streptavidin, yields substantial differences in the efficiency of the fluorescence quenching. Each of the three dyes features much more rapid decrease of the fluorescence intensity, as displayed on the lower row in Figure 2. We find 70%, 81%, and 95% of Atto 488, Atto 520, and Atto 550 molecules, respectively, are quenched after approximately 20 min. Again, similarly as for the gold nanoparticles without streptavidin, the emission is more strongly reduced for the red-shifted dyes.

**Figure 3:**
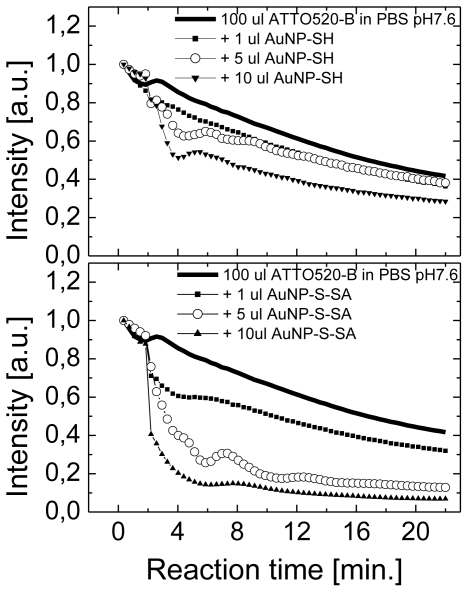
(**a**) Intensity of fluorescence measured for Atto 520 mixed with non-functionalized gold nanoparticles. (**b**) Intensity of fluorescence measured for Atto 520 mixed with functionalized gold nanoparticles. In both graphs squares, circles, and triangles correspond to 1 μL, 5 μL, and 10 μL addition of nanoparticles. Solid line represents pure Atto 520 dye. Fluorescence was excited using 485 nm laser.

The quenching dynamics on hybrid nanostructures has been shown to depend upon the amount of metallic nanoparticles added to the solution [16,18]. In Figure 3 we show an example of fluorescence intensity dependence on time of reaction for three concentrations of gold nanoparticles, 1 μL, 5 μL, and 10 μL. The experiment has been carried out for solution containing Atto 520 dye together non-functionalized gold nanoparticles (Figure 3a) and nanoparticles functionalized with streptavidin (Figure 3b). The nanoparticles were added to the solution after 2 min. After approximately 20 min. the intensity of pure dye solutions decreases by about half. In agreement with the results shown in Figure 2, addition of gold nanoparticles results in more rapid decrease of the emission and the reduction is stronger when highly concentrated gold nanoparticle solution is added to the mixture. Nevertheless, in the case of non-functionalized nanoparticles, the decrease of the emission features relatively smooth gradual characteristics for all three concentrations of gold nanoparticles. Conversely, when the nanoparticles are functionalized with streptavidin, enabling thus conjugation with the organic dyes, the decrease is much more rapid, in particular for 5 μL and 10 μL additions of gold nanoparticles. In fact, for the highest concentration of gold nanoparticles the fluorescence drops to less than 20% within the first 3 min. after the reaction. In perfect agreement, the analogous results obtained for Atto 488 have shown the fluorescence of this dye is less affected by plasmon excitations, while Atto 550 features more rapid changes in the fluorescence intensity.

Time-resolved fluorescence spectroscopy provides valuable and complementary information to the results obtained with continuous-wave laser excitation [19]. The dynamics of the fluorescence shed light into the processes responsible for the fluorescence quenching and the factors that determine its strength.

**Figure 4:**
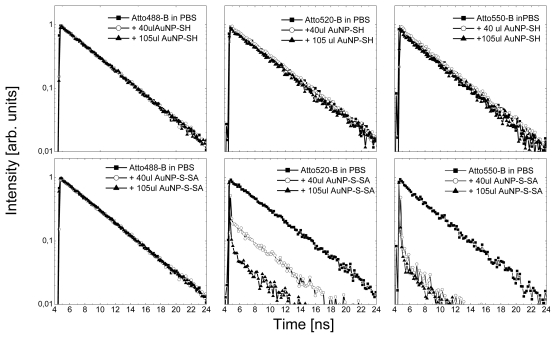
(**top row**) Normalized fluorescence transients obtained for the Atto dyes mixed with non-functionalized gold nanoparticles. Data for mixtures is compared with fluorescence decays measured for pure dye solution (solid squares). (**lower row**) Normalized fluorescence transients obtained for the Atto dyes mixed with streptavidin-functionalized gold nanoparticles. Data for mixtures is compared with fluorescence decays measured for pure dye solution (solid squares). Excitation energy of 458 nm was used for all measurements.

In Figure 4 we display fluorescence transients measured for mixtures containing organic dyes and both non-functionalized (upper row) and streptavidin-functionalized (lower row) spherical gold nanoparticles. The transients displayed were measured at the end of 20 min. long reaction, which started with adding gold nanoparticles to the solution of Atto dyes. Continuous-wave experiments indicate that for these two cases the fluorescence intensity decreases with the strongest effect observed for Atto 550. Time resolved measurements show that despite the strong fluorescence quenching observed for mixtures containing Atto dyes with non-functionalized gold nanoparticles, the fluorescence lifetime remains unchanged, even for the very high concentration of metallic nanoparticles (105 μL). The decay times of fluorescence for all three Atto dyes are very similar and equal to 4.1 ns. In contrast, the experiments carried out for mixtures of streptavidin-functionalized nanoparticles, feature strong spectral dependence of the transient behavior. In the case of Atto 488, which overlaps weakly with the plasmon resonance of the gold nanoparticles, the fluorescence lifetime remains unchanged upon addition of metallic nanoparticles to the solution. As the absorption/emission energy of the organic dyes is shifted towards longer wavelength, we observe qualitatively different behavior to all previously discussed cases. Namely, for both Atto 520 and Atto 550 mixed with streptavidin—functionalized gold nanoparticles—we find biexponential decay. The longer decay is identical to the one measured for pure dye solution, thus we attribute it to molecules that are not conjugated with gold nanoparticles, therefore not experiencing efficient non-radiative energy transfer to the metallic nanoparticle. The much faster component (~ 100 ps), which is comparable to our system resolution, we assign to the fraction of molecules that are quenched due to efficient energy transfer to the metallic nanoparticles upon conjugation.

The results shown in Figure 4 demonstrate strong influence of the spectral overlap between plasmonic resonance and the absorption/emission of the fluorophore upon the efficiency of fluorescence quenching, in full correspondence to the continuous-wave fluorescence experiment. It also shows that the mechanism responsible for decrease of the fluorescence is different in the case of functionalized gold nanoparticles, which bind with organic dyes, as compared to direct mixtures with no covalent binding taking place in the solution. The exact origin of the observed differences in the fluorescence transients is not completely clear. However, we can speculate that in the case of conjugated hybrid nanoparticles, where the separation between metallic nanoparticles and fluorescent dyes is fixed, the dominant process could be the Foerster resonant energy transfer. On the other hand, in the case of non-conjugated sample we could argue this is purely collisional quenching as the metallic nanoparticles are negatively charged and the Atto dyes are either positively (Atto 520 and Atto 550) or negatively (Atto 488) charged. The difference in charging could result in differences observed in fluorescence intensity between various dyes mixed with non-functionalized gold nanoparticles. Future experiments are planned to solve this issue. However, the strength of fluorescence quenching upon conjugating with metallic nanoparticles shows clear dependence upon the concentration of gold nanoparticles in solution. The results in Figure 4 indicate that adding 40 μL of streptavidin-functionalized gold nanoparticles to Atto 520 results in approximately 80% of molecules being conjugated, and thus quenched. Complete quenching takes place for 105 μL addition of streptavidin-functionalized gold nanoparticles. Therefore, in agreement with continuous wave fluorescence data, the results obtained for Atto 520 indicate that the relative ratio of conjugated molecules to emitting molecules increases with increasing the concentration of streptavidin-functionalized gold nanoparticles in the solution. Furthermore, for the most red-shifted Atto 550, which features the largest spectral overlap with the plasmon resonance in gold nanoparticles, the almost complete quenching of the emission takes place already upon addition of 40 μL of streptavidin-functionalized gold nanoparticles. It is not only the percentage of conjugated dyes in any given solution that depends upon the concentration of gold nanoparticles, but also the speed of the reaction itself. Namely, for the Atto 550 mixed with 20 μL of streptavidin-functionalized gold nanoparticles the intensity of dyes that are not covalently bound to the gold nanoparticles, *i.e*.,: these responsible for unchanged fluorescence decay seen in Figure 4, decreases gradually till the equilibrium is reached. The equilibrium most certainly corresponds here to the situation when all metallic nanoparticles are conjugated with the dye. In contrast, for 40 μL and 105 μL of streptavidin-functionalized gold nanoparticles the appearance of the biexponential fluorescence decay is instantaneous and the relative contribution of conjugated and non-conjugated Atto 550 molecules remains unchanged from the beginning till the end of the reaction. The results measured with pulsed excitations fully corroborate the conclusions derived from the steady-state fluorescence spectroscopy, pointing towards extremely efficient energy transfer between fluorophores and metallic nanoparticles that show large spectral overlap.

## 3. Experimental Section

### 3.1. Synthesis and Functionalization of Metallic Nanoparticles

HAuCl_4_·3H_2_O, 3-mercaptopropionic acid, L-Ascorbic Acid, PBS, Atto 488, Atto 520, and Atto 550 were obtained from Sigma-Aldrich. Streptavidin (SA) and D-biotin (B). 1-Ethyl-3-(3-dimethlamino propyl) carbodiimide hydrochloride (EDC) was obtained from Sigma-Aldrich and used bioconjugation process. NaBH_4_, CH_3_COOH were purchased from POCH S.A All reagents were purchased at highest purity and used without further purification. Deionized water (Fluka) was used in all experiments. All glassware was cleaned with pirahnia solution.

#### Preparation of Au spherical nanoparticles coated with 3-mercapropropionic acid (AuNP–S–COOH) [20]

Briefly, 1 mL of 5 × 10^−5^ M HAuCl_4_ water solution was mixed with a 34 mL of 3.25 × 10^−3^ M ethanol solution of 3-mercaptopropionic acid. A freshly prepared 0.15 M solution of NaBH_4_ (3.4 mL) was added dropwise under vigorous stirring in an ice bath to reduce the gold salt. The resulting dark brown solution was stirred for one hour after which the obtained nanoparticles were allowed to sediment to the bottom of the flask and the supernatant was removed by pipette. The particles were washed twice by dispersing them in 98% methanol and the solvent was removed by centrifugation (2000 rpm). Finally, the product was redispersed in 1 mL of water.

#### Functionalization of the gold nanoparticles with streptavidin

Protein can be directly adsorbed on gold nanoparticles via electrostatic interaction, but this method is non-specific. To make the functionalization more specific, the EDC cross-linking procedure can be applied [17].

#### Covalent binding

To link affinity bioligands on Au NPs EDC cross-linking procedure was utilized. Fresh solution of 0.2 M EDC was prepared in PBS buffer solution at pH 7.4. 50 μL of EDC solution was reacted with 2 mL of gold nanoparticles solution for 30 min. To this mixture we added 100 μL of streptavidin with a concentration of 1.6 × 10^−7^ M and left it to react for 2 h. The result of this method is a stable amide bond that is formed between free amine group of streptavidin and carboxylic group on gold nanoparticles surface.

#### Conjugation procedure

The streptavidin-biotin bond is particularly suitable for conjugating biomolecules with inorganic nanostructures, as it is one of the strongest non-covalently interacting pairs; the binding is relatively fast and only slightly affected by the pH, temperature, organic solvents, *etc*. We used the streptavidin-biotin linkage to conjugate the gold nanoparticles functionalized with streptavidin to different dyes from family Atto substituted with biotin. NPs-Atto superstructures were obtained by mixing appropriate volumes of stock solutions of NPs-SA and Atto-B. The conjugation processes were carried out in plastic UVette with a range of transparency from 220 to 1600 nm. In typical preparation of the Atto-B-SA-AuNPs conjugate, a different value of gold nanoparticles solution in range of 1 to 105 μL was added into the constant concentration of PBS water solution Atto-Biotin dyes. The following concentrations of dye molecules were used: Atto488: 5.41 × 10^−8^, Atto520: 4.1 × 10^−8^, and Atto550: 3.67 × 10^−7^. The concentration of gold nanoparticles was approximately 5.2 mM.

### 3.2. Spectroscopic Characterization of Hybrid Nanostructures in Solution

The optical properties of Atto dyes, Au nanoparticles, and mixtures thereof were investigated with UV-Vis absorption spectroscopy, fluorescence spectroscopy and time-resolved fluorescence. Absorption spectra were measured at room temperature, using quartz cuvette with a 1-cm optical path in range 350–1100 nm. Fluorescence spectra of the solutions were measured using Fluorolog 3 spectrofluorimeter (Yobin-Ivon) equipped with a photomultiplier detector and a Xenon lamp coupled to a double monochromator for the excitation. The excitation wavelength was 485 nm, which corresponds to the excitation used for time–resolved experiments. In order to evaluate the strength of fluorescence quenching upon addition of gold nanoparticles, fluorescence spectra were monitored up to 20 min. after start of the reaction.

Fluorescence transients were measured for solutions placed in cuvette. Various amount of gold nanoparticles, both non-functionalized and functionalized with streptavidin, were used. For excitation we used a laser with wavelength of 485 nm that can be operated in either continuous-wave or pulsed mode with 80 MHz repetition rate. The excitation power was 500 μW and the focal spot was about 100 μm in diameter. Fluorescence decays were collected using a Becker & Hickl SPC-150 time-correlated single photon counting card with a fast photodiode detector (idQuantique id100-50) [21]. For these measurements a set of filters comprising of FD1Y longpass and HQ550/40 (Chroma) bandpass were used. The temporal resolution of the setup was about 100 ps.

## 4. Conclusions

We studied the fluorescence properties of Atto dyes coupled to metallic nanoparticles. In order to study the influence of spectral properties of the dyes upon the fluorescence quenching caused by non-radiative energy transfer from the molecule to the nanoparticle, we used three different dyes with varied emission/absorption overlap with the plasmon resonance of gold nanoparticles. For little overlap values we observed significantly less efficient quenching of the fluorescence as compared to the strongly overlapping case, where the quenching was almost complete 20 min. after adding gold nanoparticles to the solution of organic dyes. Functionalization of the gold nanoparticles with streptavidin that enables conjugation with biotin-functionalized dyes strongly increases the efficiency of the fluorescence quenching. The results of time-resolved fluorescence spectroscopy point towards qualitatively different mechanism of fluorescence quenching for conjugated and non-conjugated mixtures of hybrid nanostructures.
